# Supplementary material for: Periodontitis promotes bacterial extracellular vesicle-induced neuroinflammation in the brain and trigeminal ganglion
Source: PLoS Pathog. 2023 Oct 23;19(10):e1011743. doi: 10.1371/journal.ppat.1011743 (PMC10621956; doi:10.1371/journal.ppat.1011743)
Supplement: S1 Table — (DOCX) [file ppat.1011743.s008.docx]

**S1 Table. Passive and active membrane properties of small-sized trigeminal ganglion (TG) neurons.**

|  | **C_m_ (pF)** | **RMP (mV)** | **R_in_ (MΩ)** | **AP amplitude**  **(mV)** | **AP duration**  **(ms)** | **AHP (mV)** | **τ of AHP**  **(ms)** | **n** |
| --- | --- | --- | --- | --- | --- | --- | --- | --- |
| Control  (PBS) | 14.9 ± 0.3 | -57.1 ± 1.5 | 691.9 ± 52.4 | 110.6 ± 2.0 | 4.6 ± 0.2 | 12.5 ± 0.9 | 54.5 ± 3.7 | 52 |
| Aa EV | 14.3 ± 0.3 | -57.4 ± 1.0 | 737.1 ± 56.4 | 111.3 ± 1.9 | 4.4 ± 0.3 | 11.6 ± 0.7 | 52.5 ± 6.2 | 50 |
| *p*-value^a^ | 0.124 | 0.855 | 0.559 | 0.807 | 0.677 | 0.413 | 0.697 |  |

Data represent the mean and SEM.

C_m_; membrane capacitance, RMP; resting membrane potential, R_in_: input resistance, AP: action potential, AHP: after hyperpolarization, τ: decay time constant

^a^unpaired t-test.
