# Supplementary material for: Periodontitis promotes bacterial extracellular vesicle-induced neuroinflammation in the brain and trigeminal ganglion
Source: PLoS Pathog. 2023 Oct 23;19(10):e1011743. doi: 10.1371/journal.ppat.1011743 (PMC10621956; doi:10.1371/journal.ppat.1011743)
Supplement: Attachment — Submitted filename: response letter_230905_RF.docx [file ppat.1011743.s009.docx]

**Response Letter to the Referees**

Thank you for submitting your work to PLOS Pathogens and I am sorry for the delay getting the reviews back to you. The Reviewers found the work of interest but there are some major issues that need to be addressed with additional experimental evidence. In particular, I agree with Reviewer 1 that specificity of the response needs to be addressed. Do other EVs have the same response as Aa EVs and are they trafficked similarly? These controls need to be demonstrated. Also, is the "dose" of EVs that are applied in this model in any way physiological? For example, how does the dose of Aa EVs compare to the concentration of bacteria (CFUs/ml) used to prepare the EVs?
These issues must addressed before further consideration of the manuscript.

**Reply:** Thank you very much for your thorough review and thoughtful considerations regarding our manuscript. Your comments have been instrumental in refining our paper. The editor's concerns have been incorporated into the responses to reviewers provided below. If allowed, we would like to make this peer-review response publicly available to enrich the understanding of both readers and the broader scientific community.

Reviewer's Responses to Questions

**Part I - Summary**

**Reviewer #1:** This research article regarding periodontal pathogen EV-induced neruoinflammation by Ha et al is an interesting and in-depth study of the ability of extracellular vesicles (EVs) from the pathogen Aggregatibacter actinomycetemcomitans (Aa) introduced via the oral route to induce inflammation in the brain in a mouse model. The authors utilize two administrative routes via the oral cavity (gingival soaking and injection) and investigate neuroinflammatory responses using signaling pathway-deficient (MyD88 KO) and immune-competent (WT) mice. They followed the trafficking of the EVs using fluorescently labeled EVs and in situ imaging. This work follows on two very closely related studies from this group that already demonstrated bEV trafficking and exRNA delivery to the brain (2020, 2019), which reduces the novel aspects of this report. The major critical areas concern dosage and specificity as detailed below. At this point in these studies, it is expected that such questions need to be addressed in order to move the field forward in a significant way.

**Reply:** Thank you for your feedback and insightful comments.

As for the novelty, we previously showed that blood-borne EVs could enter brain through blood-brain barrier and induce inflammatory cytokine production as the reviewer indicated. Here we suggest that periodontitis mediates dissemination of EVs originated from the periodontal pathogen eventually causing neuroinflammation in orofacial nerve and brain. The significant extension from early studies includes the evaluation of MyD88 pathway in the process and the use of electrophysiological methods to demonstrate transmission of EVs into sensory neurons. Furthermore, the use of ligature-induced periodontitis model, which closely resembles many aspects of human periodontitis conditions, enhances translational relevance of our findings. We believe that our study does not merely track the path of EVs, but significantly broadens our understanding of neuroinflammation and sensory neuron functionality by taking the roles of bacteria-originated EVs into account.

**Reviewer #2:** The study suggested that EVs derived from periodontopathogens such as Aa might be involved in pathogenic pathways for neuroinflammatory diseases, neuropathic pain, and other systemic inflammatory symptoms as a comorbidity of periodontitis. It was interesting and novelty.

**Reply:** We deeply appreciate your constructive feedback and recognition of the novelty in our research. We agree that our study sheds new light on the potential role of EVs derived from periodontopathogens such as Aa in the pathogenesis of neuroinflammatory diseases, neuropathic pain, and other systemic inflammatory symptoms associated with periodontitis.

This innovative perspective significantly expands our current understanding of periodontitis comorbidities, and it opens up new possibilities for investigating these systemic effects. By studying the detailed mechanisms of how Aa-derived EVs contribute to neuroinflammation and sensory neuron functionality using signaling pathway-deficient (MyD88 KO) and immune-competent (WT) mice, we hope to catalyze further investigations into effective therapeutic strategies.

Furthermore, the use of a periodontitis animal model, which closely mirrors the human condition, underscores the potential translational relevance of our work. We believe that our study paves the way for further exploration into the systemic impacts of periodontal disease and the development of effective treatment strategies.

We are encouraged by your recognition of the novelty and importance of our study, and we sincerely appreciate your insightful comments and suggestions. Your feedback will help us continue to refine our work and deepen our contribution to this important field of research.

**Part II – Major Issues: Key Experiments Required for Acceptance**

**Reviewer #1:** Major comments:
1. A serious question is the matter of physiological dosing. The authors do not address experimentally whether the EV dose they are administering via the ginigival route is at all physiologically reasonable. How does the amount of EVs entering the gingiva compare to the amount produced by Aa? Is it an amount that is absurdly high such that it would never be attained by typical Aa colonization in the gingival tissue? This study claims to test the hypothesis whether periodontitis facilitates the migration of oral bEVs to brain to induce neuroinflammation (lines 252-3), yet the experimental model is not periodontitis, it is application of purified EVs by injection or oral gel. Notably, the authors claim (lines 87-92): "Previously we showed that EVs of Aa and their RNA cargo could cross the blood-brain barrier (BBB) and increase proinflammatory cytokine expression in the mouse brain [20,21]. This was the first direct evidence that blood-borne bEVs can cross the mouse BBB and cause neuroinflammation. However, it remained unclear whether EVs originated from periodontopathogens could enter the brain through leaky gum during PD development." However, the study here does not actually address whether the EVs that are made 'during PD development' traffic in the same manner or with the same immunoinflammatory results as those that have been isolated and introduced in the quantity that they were during these experiments.

**Reply:** We sincerely appreciate your thoughtful feedback and agree that addressing the physiological relevance of the administered EV dose is a critical aspect of our study. We have estimated the approximate number of extracellular vesicles produced by the Aa bacteria, a detail now clarified in the updated manuscript.

The exact number of bacteria in the mouth at any moment is difficult to pinpoint, but it's estimated that the average adult's oral cavity contains approximately 50–100 billion bacteria, representing around 200 dominant bacterial species [1]. Additionally, one ml of saliva from a healthy adult typically contains about 100 million bacterial cells. Considering the average daily salivary flow rate is 750 ml, this translates to roughly 8 ×10^10^ bacteria being shed from the mouth's surfaces daily, which is equivalent to a wet weight of 5–10 g of bacterial cells [2].

From the Aa bacterial culture with a concentration of 5.66×10^9^ CFU/ml, we were able to purify approximately 4.5 ×10^10^ Aa EV particles/ml. This equates to about 7.9 particles per bacterial cell, though the actual number of EVs that a single bacterium can produce is likely much higher than our estimate. Given this, the dose of Aa EVs we utilized in this study –2.25 × 10^9^ particles in a 50 µl injection or gel (4.5 ×10^10^ particles/ml)– is considered to be within a physiologically reasonable range. Furthermore, the number of Aa used in our experiments are in accordance with the number of bacteria frequently used to induce periodontitis in rodents, which is between 10^9^ to 10^10^ CFU/ml [3–6].

In this updated manuscript, we have revised the entire document and standardized the numbers and volumes of Aa EV particles to avoid any confusion.

As for your concern regarding our experimental model, we concur that the application of purified EVs by injection or oral gel does not entirely replicate the conditions of periodontitis since Aa bacteria normally reside within the biofilm and our experimental model may not fully replicate this condition. However, at least we could show that when Aa EVs are in the gingiva (inoculation) or in contact with tooth and periodontium (gel application) the disruption of epithelial barrier by induction of periodontitis (ligature) could mobilize Aa EVs into brain and induce inflammation.

In the current experiments we did not track EVs produced during periodontitis development *in situ* but investigated those previously isolated and introduced into mice. It is partly due to that periodontitis does not naturally occur in mice and Aa bacteria are not naturally present in the oral environment of these animals [7]. Although the induction of periodontal disease in the mice by ligature placement and administration of Aa EVs were performed in a manner as closely aligned with human conditions as possible, we have to admit that there are inherent limitations. In response to the reviewer's comment, we have made adjustments to the wording in the current version of the manuscript. The revised statement now reads: “However, it remains unclear whether EVs originating from periodontopathogens can enter the brain through leaky gum in humans with PD.”

Our previous work demonstrated that blood borne Aa EVs and their RNA cargo could cross BBB and induce neuroinflammation. Our current study extends this understanding by suggesting that EVs present in oral environment can gain access to the brain when epithelial barriers are destroyed by the induction of periodontitis. While we recognize that our current model does not capture all complexities of periodontitis, we believe it provides a necessary foundation for studying the potential neuroinflammatory effects of periodontopathogen-derived EVs.

We hope these clarifications and additions address your concerns and thank you for your insightful comments which have undoubtedly helped strengthen our work.

2. Another critical question that is not addressed by these experiments is specificity. Obviously, there are numerous commensal species and strains of Gram-negative oral bacteria other than Aa which are also making EVs. Why would those EVs not traffic similarly through the BBB and generate neuroinflammatory disease? Such qualifications should also be considered to modify statements such as (Lines 349-351) "These findings suggest certain inflammatory disorders may be associated with the translocation of bEVs to systemic organs. Therefore, the ability of bEVs to circulate throughout the body may help in understanding autoimmune disorders, up to 50% of which lack a definite etiological cause [73]." There must be much more to generate specificity of transport and overcoming barriers in tissues, otherwise all tissues and sites would be flooded with bEVs that were produced by the human microbiome and all would cause pathologies due to PAMPs carried by the bEVs.

**Reply:** We thank you for raising the crucial point about specificity. You are correct in highlighting the multitude of oral bacteria that produce EVs and questioning their potential to cross the BBB and incite neuroinflammatory disease.

Our study specifically focused on EVs derived from Aa due to its established role as a significant periodontal pathogen and its well-documented functions in systemic diseases. While other commensal bacteria certainly produce EVs, the specific contribution of Aa-derived EVs to neuroinflammation has not been thoroughly explored.

However, we fully agree that a comprehensive understanding of the influence of EVs from diverse oral bacteria on neuroinflammation is critical.

We agree that the trafficking of bEVs to the brain may not be limited to those derived from Aa. Since our first demonstration that Aa EVs can cross the BBB and reach the brain, subsequent studies have provided supporting evidence. For example, extracellular vesicles from the gram-positive oral bacterium *Filifactor alocis* were detected in the mouse brain following intraperitoneal injection [8]. Similar observations have been made with *Helicobacter pylori* EVs, which can also cross the BBB and reach the brain [9]. In this reply letter, we offer additional evidence, showing that EVs from gram-negative bacteria such as *E. coli* and *Porphyromonas gingivalis*, as well as from the gram-positive bacterium *Lactobacillus reuteri*, can reach the brain when applied in an EV-soaked gel in a mouse model of PD (Fig R1). These findings suggest that the brain is not an isolated organ, protected from bacterial EVs. In cases where leakage occurs, bEVs and their cargo can infiltrate the brain. This might offer a clue as to why post-mortem analyses have shown increased bacterial populations in Alzheimer's disease brain tissue compared to normal brain tissue [10]; it is possible that bEVs deliver bacterial genes to the brain continuously.

As we suggested in S1 Fig of the *Supporting Information*, “Depending on the organ, it may be possible for it to serve as both a target and an elimination organ. Most Aa EVs accumulate in the spleen and liver but induce weaker proinflammatory cytokine release, suggesting that the liver and spleen might act as elimination organs for blood-borne bEVs and their cargo, including RNAs,” we believe that if leakage occurs, bEVs can enter the bloodstream at any time. However, the host immune system is also equipped to actively eliminate these bEVs and PAMPs originating from bacteria. Monocytes in various organs—and possibly microglial cells in the brain—may be involved in this elimination process. In this study, we examined the relatively short-term effects of bEVs; the elevated levels of proinflammatory cytokines would not be sustained for a long time by a single injection of bEVs. We are currently investigating the long-term effects of bEVs in relation to neuroinflammation and aim to present these findings in a future paper.

We appreciate your insight, and have addressed these points in the *Discussion* section of our revised manuscript. It now includes the following statement: “These findings suggest certain inflammatory disorders may be associated with the translocation of bEVs to systemic organs. Therefore, the ability of bEVs to circulate throughout the body may clarify our understanding of autoimmune disorders, for which up to 50% lack a definite etiological cause, while it should be noted that the majority of bEVs can be eliminated in the spleen and liver, as shown in S1 Fig.”

Thank you for your constructive critique; it has unquestionably helped to enrich our work.

**Fig R1.** EVs isolated from various bacteria can be crossed blood-brain barrier in a mouse model of ligature-induced periodontal disease (PD) using EV-soaked gel. Brain tissues were isolated to measure the accumulated fluorescence signal of DiD-labeled Aa EVs using an in vivo imaging system (IVIS) in the brain. (A) Gelatin gels (50 µl) including DiD-labeled *Escherichia coli* EVs (total 7 × 10^8^ particles) were administered to mice. *E. coli* EV signals were captured and quantified using IVIS at 24 h (n = 7 per each group) of EV-soaked gel treatments. (B) Gelatin gels (50 µl) including DiD-labeled *Porphyromonas gingivalis* EVs (total 3.1 × 10^8^ particles) were administered to mice. *P. gingivalis* EV signals were captured and quantified using IVIS at 24 h (n = 7 per each group) of EV-soaked gel treatments. (C) Gelatin gels (50 µl) including DiD-labeled *Lactobacillus reuteri* EVs (total 2 × 10^10^ particles) were administered to mice. *L. reuteri* EV signals were captured and quantified using IVIS at 24 h (n = 7 per each group) of EV-soaked gel treatments.

**Reviewer #2:** 1. Why choose 24h for osteoclastic activity?

**Reply:** Thank you for raising this insightful question. We chose to examine osteoclast activity at the 24-h mark to align with the timing of animal sacrifice in all subsequent experiments after ligation. The experiment illustrated in Figure 2B aimed to confirm the onset of periodontal disease following ligature placement. Periodontitis leads to a variety of effects, including epithelial barrier disruption, inflammatory cell infiltration, cytokine production, soft tissue destruction, and bone resorption by osteoclasts. Most of these manifestations become readily detectable within 3 to 7 days post-ligation [11–13]. We could detect osteoclast activity as an early indicator of periodontal disease, observable as early as 24 h after ligation. It's worth noting that full osteoclast activation typically requires 3 to 7 days.

2. Line 949-950, After 24 h, gingival tissues were stained for TRAP activity, the representative images of TRAP-positive cells 951 being shown (scale bar: 200 μm). It was unreasonable to analyse TRAP staining in gingival tissues.

**Reply**: Thank you for bringing this important oversight to our attention. As indicated in the *Results* section of the main text, osteoclasts were observed in the alveolar bones. In accordance with this, we have updated the term 'gingival tissues' to 'periodontal tissues' in the legend of Figure 2.

**Part III – Minor Issues: Editorial and Data Presentation Modifications**

**Reviewer #1:** (No Response)

**Reviewer #2:** 1.Lack of animal ethics approval number.

**Reply:** We fully understand the reviewer's concerns. The animal ethics approval number is located in the Ethics Statement section, found at the end of the Materials and methods.

2.How to exclude the influence of unilateral molding on the opposite side?

**Reply:** Thank you for your thoughtful question. In our study, we showed that bacterial EVs can be introduced into the brain via the bloodstream. To simulate this, we used a mouse model of periodontal disease induced by ligature, providing a pathway to the bloodstream. We also created an EV-soaked gel to closely replicate the conditions experienced by periodontal patients. This allows for the gradual release of bacterial EVs into the bloodstream. We refrained from using bilateral ligature as it would interfere with the mice's ability to feed. By employing a sham control—where no condition of periodontal disease was induced through ligature—we are confident that we have effectively excluded the impact of unilateral molding on the opposite side.

3.The image resolution was low.

**Reply:** Apologies for the inconvenience. The low image resolution was a result of PDF conversion, but we believe the original images should be downloadable. To address this issue, we have uploaded our image files to the Preflight Analysis and Conversion Engine (PACE) digital diagnostic tool to ensure they meet *PLOS Pathogens* requirements in the updated manuscript.

4.Quantitative statistics are required for positive TRAP staining cells.

**Reply:** Thank you for your insightful comments. Qualitative statistics results have been added to Figure 2 in the updated version of the manuscript.

5.p value should be in italics.

**Reply:** Thank you for your insightful comments. The issues raised by the reviewer have been addressed in the current version of the manuscript.

6.*p≤0.05 was wrong.

**Reply:** Thank you for your astute observations. The concerns highlighted by the reviewer have been resolved in the updated version of the manuscript.

7.Scale bar was missing in Fig 6. D.

**Reply:** We sincerely apologize for any inconvenience caused. The scale bar in Figure 6D was originally positioned in the upper left region, but we have relocated it to the lower right corner to maintain consistency across all figures.

8.The volume of injected Aa EVs and Aa EV-soaked gel should be demonstrated.

**Reply:** Thank you for bringing attention to this crucial aspect. In our study, we either injected 4.5 × 10^10^ Aa EV particles in 50 µl of PBS into the gingiva of the left maxilla or applied 50 µl of Aa EV-soaked hydrogel containing the same amount of EV particles to the ligated area in the periodontium (or the corresponding region in control mice). Further details on hydrogel preparation are elaborated in the *Materials and Methods* section of the updated manuscript.

**References**

1.	Krishnan K, Chen T, Paster B. A practical guide to the oral microbiome and its relation to health and disease. Oral Dis. 2017;23: 276–286. doi: 10.1111/odi.12509.

2.	Curtis MA, Zenobia C, Darveau RP. The Relationship of the Oral Microbiotia to Periodontal Health and Disease. Cell Host Microbe. 2011;10: 302–306. doi: 10.1016/j.chom.2011.09.008.

3.	Monasterio G, Budini V, Fernández B, Castillo F, Rojas C, Alvarez C, et al. IL-22-expressing CD4+ AhR+ T lymphocytes are associated with RANKL-mediated alveolar bone resorption during experimental periodontitis. J Periodontal Res. 2019;54: 513–524. doi: 10.1111/jre.12654.

4.	Ancuta DL, Alexandru DM, Crivineanu M, Coman C. Induction of periodontitis using bacterial strains isolated from the human oral microbiome in an experimental rat model. Biomedicines. 2023;11: 2098. doi: 10.3390/biomedicines11082098.

5.	Monasterio G, Castillo F, Ibarra JP, Guevara J, Rojas L, Alvarez C, et al. Alveolar bone resorption and Th1/Th17-associated immune response triggered during Aggregatibacter actinomycetemcomitans-induced experimental periodontitis are serotype-dependent. J Periodontol. 2018;89: 1249–1261. doi: 10.1002/JPER.17-0563.

6.	Monasterio G, Castillo F, Astorga J, Hoare A, Terraza-Aguirre C, Cafferata EA, et al. O-polysaccharide plays a major role on the virulence and immunostimulatory potential of *Aggregatibacter actinomycetemcomitans* during periodontal infection. Front Immunol. 2020;11: 591240. doi: 10.3389/fimmu.2020.591240.

7.	Joseph S, Aduse-Opoku J, Hashim A, Hanski E, Streich R, Knowles SCL, et al. A 16S rRNA gene and draft genome database for the murine oral bacterial community. mSystems. 2021;6: e01222-20. doi: 10.1128/mSystems.01222-20.

8.	Kim HY, Song M-K, Gho YS, Kim H-H, Choi B-K. Extracellular vesicles derived from the periodontal pathogen Filifactor alocis induce systemic bone loss through Toll-like receptor 2. J Extracell Vesicles. 2021;10: e12157. doi: 10.1002/jev2.12157.

9.	Xie J, Cools L, Van Imschoot G, Van Wonterghem E, Pauwels MJ, Vlaeminck I, et al. Helicobacter pylori‐derived outer membrane vesicles contribute to Alzheimer’s disease pathogenesis via C3‐C3aR signalling. J of Extracellular Vesicle. 2023;12: 12306. doi: 10.1002/jev2.12306.

10.	Emery DC, Shoemark DK, Batstone TE, Waterfall CM, Coghill JA, Cerajewska TL, et al. 16S rRNA Next Generation Sequencing Analysis Shows Bacteria in Alzheimer’s Post-Mortem Brain. Front Aging Neurosci. 2017;9: 195. doi: 10.3389/fnagi.2017.00195.

11.	Kim Y, Kim MO, Kim S, Kim HJ, Pokhrel NK, Lee JH, et al. 6‐Shogaol, an active ingredient of ginger, inhibits osteoclastogenesis and alveolar bone resorption in ligature‐induced periodontitis in mice. J Periodontol. 2020;91: 809–818. doi: 10.1002/JPER.19-0228.

12.	Abe T, Hajishengallis G. Optimization of the ligature-induced periodontitis model in mice. J Immunol Methods. 2013;394: 49–54. doi: 10.1016/j.jim.2013.05.002.

13.	Siddiqui YD, Nie X, Wang S, Abbasi Y, Park L, Fan X, et al. Substance P aggravates ligature-induced periodontitis in mice. Front Immunol. 2023;14: 1099017. doi: 10.3389/fimmu.2023.1099017.
